# Exercise Improves Dermal Collagen and Elastic Fibers: In Vivo Evidence From Female Volunteers and an Animal Model

**DOI:** 10.1111/jocd.71237

**Published:** 2026-10-21

**Authors:** Qi Luo, Minghao Wang, Zhu Shen

**Affiliations:** ^1^ School of Medicine South China University of Technology Guangzhou Guangdong China; ^2^ Department of Dermatology Guangdong Provincial People's Hospital, Guangdong Academy of Medical Sciences, Southern Medical University Guangzhou Guangdong China

**Keywords:** collagen fibers, elastic fibers, exercise, noninvasive assessment, skin rejuvenation, two‐photon microscopy

## Abstract

**Background:**

With increasing interest in aesthetic appearance and skin health, direct, objective, in vivo evidence remains limited regarding whether exercise improves the structural status of dermal collagen and elastic fibers.

**Objective:**

To investigate the effects of exercise on dermal collagen and elastic fiber status.

**Methods:**

This study combined an animal experiment with a human observational study. In the animal experiment, healthy female C57BL/6J mice were divided into a middle‐aged exercise group (ME, *n* = 10, 43 weeks old), a middle‐aged sedentary group (MS, *n* = 5, 43 weeks old), and a young sedentary group (YS, *n* = 5, 6–8 weeks old). The ME group underwent 4 weeks of voluntary wheel running, whereas the MS and YS groups served as sedentary controls representing different age stages. After 4 weeks, in vivo two‐photon imaging of dorsal skin was performed in all three groups. Two‐photon images were quantitatively analyzed to obtain autofluorescence (AF), second harmonic generation (SHG), the SHG‐to‐AF aging index of dermis (SAAID), and the elastin‐to‐collagen ratio (ELCOR). Immunofluorescence staining for Collagen I and Elastin was performed on skin tissue from the imaging site. In the human study, a total of 156 female volunteers completed an exercise questionnaire and underwent two‐photon imaging of the right zygomatic region of the face. SHG, AF, SAAID, and ELCOR were obtained using an image analysis approach consistent with that used in the animal experiment. Weekly total exercise duration (WeeklyMinutes) was calculated from the questionnaire. Participants were analyzed using both a 150 min/week grouping strategy and a median‐based grouping strategy. Propensity score matching was first performed using age as the covariate, followed by multivariable regression adjustment in the matched samples.

**Results:**

In the animal experiment, SAAID, SHG, and AF were increased and ELCOR was decreased in the ME group compared with the MS group, with several indicators shifting toward YS levels. Immunofluorescence showed stronger Collagen I‐ and Elastin‐related signals in the ME group than in the MS group, consistent with the two‐photon results. In the human study, 48 and 69 matched pairs were obtained after propensity score matching under the 150 min/week and median‐based grouping strategies, respectively. The results were consistent across both grouping strategies. Higher exercise levels were associated with higher SAAID and SHG, lower ELCOR, and no significant difference in AF. After further adjustment for BMI, smoking, alcohol use, and average daily sleep duration, the overall pattern of the results remained unchanged.

**Conclusion:**

Using two‐photon microscopy, this study provides direct, objective, in vivo evidence that exercise may favorably affect dermal collagen and elastic fibers. These findings provide experimental and population‐based evidence supporting exercise as a strategy for maintaining skin health.

**Trial Registration:**

This study was registered in the Chinese Clinical Trial Registry (registration number: ChiCTR2600123115)

## 1 Introduction

Collagen and elastic fibers are essential for maintaining skin tension, elasticity, and structural integrity [1, 2]. With aging, these fibers may decrease in quantity, become disorganized or fragmented, and lose function, leading to skin laxity, reduced elasticity, and wrinkle formation [3, 4]. Therefore, the status of collagen and elastic fibers is not only an important structural basis of skin aging [5], but also an important indicator for evaluating skin rejuvenation [6].

With increasing public interest in aesthetic appearance and skin health, strategies for maintaining healthy skin have attracted growing attention. Current skin rejuvenation interventions mainly include topical agents, laser treatments, and injectable treatments. Topical agents, such as retinoids, peptides, and antioxidants, are common local management approaches, but their effects usually depend on long‐term and regular use [7]. Laser treatments can help induce skin remodeling and improve skin quality, but they rely on professional equipment, usually require multiple treatment sessions, and may be limited by recovery time, erythema, or pigmentation [8]. Injectable treatments are also commonly used for skin rejuvenation, but their effects are often temporary, usually require regular maintenance, and involve considerations of injection‐related risks and financial burden [9]. Exercise is a low‐cost and sustainable lifestyle intervention with systemic health benefits. It has been widely studied in relation to metabolism, cardiovascular health, and the musculoskeletal system, but direct in vivo imaging evidence of its effects on deeper skin structures, especially collagen and elastic fibers, remains limited [10]. Previous studies have suggested that regular exercise may be associated with better skin condition [11], but this association still lacks direct, objective, in vivo evidence at the level of dermal fibers.

Two‐photon microscopy provides a new approach to this question. Based on two‐photon excited fluorescence and second harmonic generation, this technique can noninvasively obtain structural information from deeper skin layers [12, 13]. Quantitative analysis of two‐photon images can extract four indicators, including autofluorescence (AF), second harmonic generation (SHG), the SHG‐to‐AF aging index of dermis (SAAID), and the elastin‐to‐collagen ratio (ELCOR), to evaluate collagen‐ and elastic fiber‐related status [14]. Two‐photon microscopy offers several advantages, including avoidance of biopsy, repeatability, and in vivo observation [15].

At present, in vivo, visual, and objective imaging evidence remains insufficient to clarify the actual effect of exercise on dermal collagen and elastic fibers. Therefore, this study combined animal experiments with a human population study and used two‐photon microscopy to noninvasively assess microscopic indicators related to skin collagen and elastic fibers in vivo. The aim was to systematically investigate the effect of regular exercise on the status of dermal collagen and elastic fibers and to provide scientific evidence and experimental support for exercise as a safe, economical, and sustainable strategy for skin health management.

## 2 Materials and Methods

### 2.1 Overall Study Design

This study included two components: an animal validation experiment and a human observational study. The animal component was a controlled intervention experiment designed to observe changes in two‐photon indicators and histological expression of collagen and elastic fibers in mouse skin after 4 weeks of exercise intervention. The human component was a cross‐sectional observational study designed to analyze the association between daily exercise levels and two‐photon indicators of female facial skin. Weekly total exercise duration (WeeklyMinutes) was used as the core exercise exposure indicator in the human study. Analyses were performed using both a 150 min/week grouping strategy and a median‐based grouping strategy. Before between‐group comparisons, propensity score matching was performed using age as the covariate, and BMI, smoking, alcohol use, and average daily sleep duration were further included for multivariable regression adjustment in the matched samples.

### 2.2 Animal Experimental Design

The overall protocol was approved by the Ethics Committee of Guangdong Provincial People's Hospital (approval No. KY2026‐119‐01). Healthy female C57BL/6J mice were divided into three groups according to age and exercise intervention: the middle‐aged exercise group (ME, *n* = 10), middle‐aged sedentary group (MS, *n* = 5), and young sedentary group (YS, *n* = 5). The YS group consisted of 6–8‐week‐old mice, whereas the MS and ME groups consisted of 10‐month‐old mice (approximately 43 weeks old). As an exploratory preclinical study, the animal sample size was determined based on experimental feasibility and the need to obtain preliminary in vivo imaging and histological evidence. A larger sample size was allocated to the ME group to better estimate exercise‐related variation, while the MS and YS groups served as sedentary control groups. There were no significant baseline differences in body weight or general condition between the MS and ME groups. Housing temperature, humidity, light–dark cycle, diet, and water supply were kept consistent among the three groups.

Mice in the ME group were housed with a voluntary running wheel device (Representative mouse exercise activity is provided in Video S1) and underwent 4 weeks of intervention. The number of wheel rotations was recorded daily and converted into running distance to reflect actual exercise level. The YS and MS groups were maintained under the same housing conditions without additional exercise intervention and served as sedentary and physiological controls under different age backgrounds. Voluntary wheel running rather than forced exercise was used mainly to reduce stress and to better mimic spontaneous daily activity.

At the end of the experimental period, dorsal skin from each group was examined by in vivo two‐photon imaging, and tissue from the same site was collected for fluorescence staining to validate the histological changes corresponding to the two‐photon results.

### 2.3 In Vivo Two‐Photon Imaging of Mouse Skin

After 4 weeks of exercise intervention, in vivo two‐photon imaging was performed on the dorsal skin of mice in all three groups. The system used was a commercially available in vivo two‐photon microscopy imaging system (SV780, Nanjing Transcend Vivoscope Bio‐Technology Co. Ltd., Nanjing, China), which differs from a conventional laboratory benchtop two‐photon microscope. The system mainly consisted of a main unit and a handheld flat‐contact probe. The handheld probe was connected to the main unit through a flexible optoelectronic composite cable, and its front end had a flat optical window. During imaging, the probe was placed in direct contact with the body surface and could perform high‐resolution three‐dimensional optical sectioning of different skin surface sites. Based on two‐photon excited fluorescence (TPEF) and second harmonic generation (SHG), this system enables in vivo, in situ, noninvasive, label‐free, real‐time imaging of the skin. The excitation wavelength of the system was 780 ± 10 nm, with a laser output power of 50 mW±10%. The pulse repetition rate was 80 MHz±10%, and the pulse width did not exceed 200 fs. Two detection channels were used in this study: 420–580 nm for the AF channel and 375–410 nm for the SHG channel. The scanning field of view was no less than 125 μm × 125 μm, and the lateral resolution was no greater than 0.8 μm. The system imaging depth parameter was no less than 200 μm. Three‐dimensional fast scanning mode was used, with a raw frame rate of 9 fps.

For all animals, before imaging, a dorsal skin area of approximately 2 × 5 cm between the scapular and lumbosacral regions was clipped and depilated. The imaging site was uniformly selected from the relatively flat central area of this treated region, while avoiding skin folds, residual hair, local damage, and areas where stable probe contact could not be maintained. Skin tissue was collected from the corresponding imaged site for subsequent fluorescence staining. This site‐selection procedure was kept consistent across all groups to reduce the interference of structural differences among different dorsal skin regions on two‐photon signals. Mice were anesthetized with inhaled isoflurane. The dorsal detection area was clipped with an electric animal clipper, followed by the use of depilatory cream to remove residual hair, and any remaining depilatory cream on the skin surface was removed. The detection area was then gently cleaned with alcohol, and the handheld probe of the in vivo two‐photon microscopy imaging system was placed on this site for image acquisition. No additional coupling medium was used during imaging.

During image acquisition, the stratum corneum was used as a unified zero‐depth reference, and continuous optical sectioning was performed from the stratum corneum with a *z*‐step interval of 2 μm to control systematic bias in two‐photon signals caused by differences in imaging depth. The actual acquisition depth in mouse dorsal skin was 40–45 μm. Serial optical sections showed that collagen‐related SHG signals and elastic‐fiber‐related AF signals were relatively clear and stable in the superficial dermis approximately 10–20 μm below the stratum corneum. Based on this observation, this depth range was defined as the quantitative analysis window for mouse SAAID and ELCOR. Quantitative analysis of all mouse samples was restricted to this window. Consecutive optical sections were included in the analysis only when they met all of the following criteria: AF and SHG channel signals were clearly identifiable, the images showed no obvious defocusing, motion artifacts, or signal saturation, and the field of view was not affected by obvious skin folds, local damage, or large skin appendage structures. The same optical‐section selection criteria were applied to all groups.

Endogenous components in the skin, including NAD (P) H, FAD, elastic fibers, melanin, and keratin, can generate fluorescence signals under two‐photon excitation, whereas collagen fibers in the dermis generate strong second harmonic signals [16]. In this study, AF (autofluorescence) specifically refers to endogenous autofluorescence signals acquired through the two‐photon excitation channel, namely the TPEF signals collected in the AF channel. TPEF denotes the excitation mechanism and signal type, while AF denotes the autofluorescence channel used in subsequent analyses. In the analytical context of this study, the AF channel mainly reflects elastic fiber‐related autofluorescence signals, while the SHG channel mainly reflects collagen fiber‐related signals [15, 17].

After image acquisition, AF channel images, SHG channel images, and merged AF–SHG images were saved for subsequent analysis [18]. According to the analysis indicators defined by the system software, SHG, AF, SAAID, and ELCOR were extracted. SAAID (SHG‐to‐AF aging index of dermis) [19] characterizes the normalized relationship between SHG and AF signals and is classically defined as SAAID = (SHG − AF)/(SHG + AF). ELCOR (elastin‐to‐collagen ratio) [20] represents the relative ratio between elastic fiber‐related signals and collagen fiber‐related signals.

SAAID is an optical index related to dermal aging that is calculated based on collagen‐related SHG signals and AF signals and reflects the relative balance between SHG and AF signals in the dermis. SHG signals mainly originate from fibrillar collagen with a non‐centrosymmetric structure, whereas AF signals in the dermis are mainly associated with elastic fibers and other endogenous fluorescent components. Therefore, SAAID can be used as an optical parameter reflecting changes in collagen‐related signals relative to AF signals in the dermis [19]. Human multiphoton imaging studies have shown that SAAID is generally negatively correlated with age, with higher values usually indicating a higher proportion of collagen‐related SHG signals relative to AF signals and a more youthful dermal fiber status. This index has been used to evaluate intrinsic skin aging, photoaging, and structural changes related to the dermal extracellular matrix [21, 22].

ELCOR reflects the relative proportion between elastic‐fiber‐related AF signals and collagen‐related SHG signals, and can be used to describe relative compositional changes between elastic fibers and collagen fibers in the dermis [20]. ELCOR, or related dermal fiber composition ratio indices based on AF and SHG signals, has also been used to evaluate changes in the dermal extracellular matrix associated with age, skin depth, photoexposed sites, and different body sites [23]. It should be noted that there are currently no widely accepted normal reference ranges or standardized cut‐off values for SAAID or ELCOR. The values of these indices may be affected by detection site, imaging depth, device parameters, image processing method, threshold setting, and selection of the analysis region [17, 24]. In this study, SAAID and ELCOR were used as relative quantitative indices for between‐group comparisons under the same imaging conditions and analysis workflow, rather than as absolute indicators for clinical diagnosis, individual classification, or threshold‐based interpretation.

For each mouse, three qualified consecutive optical sectioning segments were selected within the above superficial dermal analysis window. Specifically, the section in which elastic fibers were relatively extended and both collagen‐related SHG signals and elastic‐fiber‐related AF signals were clearly identifiable was selected as the starting section. The following consecutive 3–5 optical sections were then included as one analysis segment, provided that the fiber morphology remained clear throughout this range. SHG, AF, SAAID, and ELCOR were extracted from each segment, and the mean of the three measurements was used as the final result for that animal.

### 2.4 Immunofluorescence Staining Analysis of Mouse Skin Tissue

After in vivo two‐photon imaging of mouse dorsal skin was completed, mice were immediately euthanized by carbon dioxide inhalation, and skin tissue from the same site was collected for subsequent fluorescence staining. The staining markers included Collagen I, Elastin, and DAPI. Collagen I signal was displayed in the Cy3 channel, Elastin signal was displayed in the FITC channel, and DAPI was used for nuclear counterstaining. HE staining images were also obtained as a morphological reference but were not included in subsequent quantitative analysis. Fluorescence‐scanned section images were analyzed using Fiji/ImageJ (64‐bit) [25].

For quantitative analysis, the dermal fiber region was used as the main observation area. Each section was systematically sampled along the dermal length. The analyzable dermal band was roughly divided into five consecutive segments, and one region of interest (ROI) was selected from each segment, resulting in five ROIs per section that covered the entire section as much as possible. ROIs were drawn as band‐shaped polygons with the long axis aligned as closely as possible with the dermal direction, and the area was controlled within 20 000 ± 10% μm^2^ (18000–22 000 μm^2^). During ROI selection, DAPI and merged images were used to assist in identifying tissue layers and adnexal structures. Areas with clear dermal structure and relatively uniform distribution were prioritized, and the selected regions were kept at a relatively consistent depth. Any ROI that clearly contained epidermis, hair follicles, sebaceous glands, adnexal structures, subcutaneous fat, vacuolated regions, fissures, folds, tissue damage, edge artifacts, or obvious abnormal structures surrounding vessels or lumens was considered unqualified. In such cases, another ROI was selected within the same segment.

The quantitative indicator was positive area percentage (Area%), defined as the percentage of ROI area occupied by pixels identified as positive signal after thresholding. Collagen I Area% reflected the coverage proportion of Collagen I‐positive signal, and Elastin Area% reflected the coverage proportion of Elastin‐positive signal. Threshold segmentation was performed using a unified automatic threshold that was kept consistent across all samples. After obtaining Area% from five ROIs per section, the mean value was calculated as the result for each animal. For between‐group comparisons, each mouse was used as the statistical unit, and ROIs served only as systematic sampling units within the same animal. All images were quantified by the same analyst according to uniform rules, and group information was blinded during analysis.

### 2.5 Human Participants and Facial Two‐Photon Detection

The human study protocol was approved by the Ethics Committee of Guangdong Provincial People's Hospital (approval No. KY2026‐119‐01). All participants provided written informed consent before enrollment. The study was registered in the Chinese Clinical Trial Registry (registration No. ChiCTR2600123115). In this preliminary observational study, only female participants were recruited via open enrollment. Females exhibit higher compliance with skin examinations and maintain more consistent skincare routines and lifestyle habits, which helps reduce confounding bias. Inclusion criteria were female sex, age 18–60 years, voluntary participation, completion of facial two‐photon imaging, and completion of the exercise questionnaire. Exclusion criteria included missing age or exercise questionnaire information, inability to obtain qualified continuous analyzable image frames from the target detection area, active skin disease, or an obvious scar or pigmented nevus at the measurement site that could affect optical imaging. Participants were also excluded if they had received light‐ or energy‐based aesthetic treatment or chemical peeling at the site within the past 3 months, or were currently using systemic glucocorticoids or immunosuppressive drugs that may affect skin structure. The participants included in this study were ordinary female volunteers and did not include professional athletes, competitive athletes, or individuals who had received long‐term systematic professional athletic training. The differences in exercise level in the human component mainly reflected differences in daily exercise habits among ordinary women rather than differences between athletes and non‐athletes.

Facial skin detection in humans was performed using the same in vivo two‐photon microscopy system as that used in the animal experiment [26]. Before detection, the examined area was cleaned and wiped with alcohol. All measurements were performed at the same room temperature by the same operator, and the detection site was uniformly selected as a relatively flat region of the right zygomatic area. During image acquisition, the stratum corneum was used as the zero‐depth reference, and continuous optical sectioning was performed from the stratum corneum with a *z*‐step interval of 2 μm. The actual acquisition depth in the facial skin of female participants was 0–100 μm. Based on inspection of the original images, collagen‐related SHG signals and elastic‐fiber‐related AF signals were relatively clear and stable within the depth range of 50–90 μm. This depth range was therefore used as the main quantitative analysis window for human SAAID and ELCOR. For each participant, one qualified continuous optical sectioning segment was selected within this analysis window. The starting optical section was defined as the section in which elastic fibers were relatively extended and both collagen‐related SHG signals and elastic‐fiber‐related AF signals were clearly identifiable. The ending section was set within the following consecutive 3–5 optical sections, provided that the fiber morphology remained clear throughout this range. SHG, AF, SAAID, and ELCOR were then extracted as the final results for that participant.

### 2.6 Exercise Questionnaire and Definition of WeeklyMinutes and Grouping

In the human study, weekly total exercise duration (WeeklyMinutes) was used as the core exercise exposure indicator. FreqDays and MinPerSession were calculated from two questionnaire items. These items assessed the number of days per week on which participants engaged in exercise lasting more than 10 min and causing sweating or a noticeable increase in breathing or heart rate, and the average duration of each exercise session on exercise days. Weekly exercise frequency was converted as follows: 0 days, 1–2 days, 3–4 days, and ≥ 5 days were assigned values of 0, 1.5, 3.5, and 5.5 days, respectively. Exercise duration per session was converted as follows: < 30 min, 30–60 min, and > 60 min were assigned values of 20, 45, and 75 min, respectively. WeeklyMinutes was then calculated using the formula WeeklyMinutes = FreqDays × MinPerSession. The self‐designed exercise questionnaire was developed with reference to adult physical activity guidelines and was reviewed by clinical experts to improve its content validity and comprehensibility.

Two predefined exercise exposure definitions were used in the population study. The first was based on the recommendation in adult physical activity guidelines [27] of at least 150 min per week of moderate‐intensity aerobic physical activity, and participants were divided into ≥ 150 min/week and < 150 min/week groups as the primary analysis strategy [28]. The second was based on the median WeeklyMinutes in this cohort, and participants were divided into an above‐median group and an at‐or‐below‐median group as a supplementary analysis strategy.

### 2.7 Propensity Score Matching

To reduce the potential confounding effect of age on skin two‐photon indicators in the population study, propensity score matching (PSM) was used to balance groups [29, 30]. Age was used as the covariate, and a logistic regression model was used to estimate each participant's propensity score for being in the high‐exercise group. Matching was performed using 1:1 nearest‐neighbor matching without replacement. The caliper was set at 0.2 times the standard deviation of the logit of the propensity score [31]. The same matching approach was applied to both the 150 min/week and median‐based grouping analyses. After matching, improvement in covariate balance was assessed by comparing standardized mean differences before and after matching. Subsequent between‐group comparisons of SHG, AF, SAAID, and ELCOR were performed based on the matched samples. Because BMI, smoking, alcohol use, and average daily sleep duration may still be imbalanced after matching, these variables were further included in multivariable regression adjustment in the matched samples [32].

### 2.8 Statistical Analysis

In the mouse experiment, each indicator was analyzed using each mouse as the statistical unit, and continuous variables were presented as mean ± standard deviation. Before analysis, data distribution and homogeneity of variance were assessed. When parametric assumptions were met, one‐way ANOVA was used to compare differences among the three groups, and Tukey's test was used for pairwise comparisons. If parametric assumptions were not met, the Kruskal–Wallis test and corresponding multiple comparisons were used. Statistical analysis was performed using GraphPad Prism 10.1.2.

Human data were analyzed in the propensity‐score‐matched samples. Matched data were treated as paired data. Depending on the distribution of paired differences, paired *t*‐tests were used when the differences were approximately normally distributed; otherwise, Wilcoxon signed‐rank tests were used. Continuous variables were presented as mean ± standard deviation or median (interquartile range). Covariate balance was described using standardized mean differences (SMD), with |SMD| < 0.1 generally considered to indicate good balance between groups [33]. All statistical tests were two‐sided, and *p* < 0.05 was considered statistically significant. For the four main two‐photon outcomes in the human study, namely SHG, AF, SAAID, and ELCOR, the Benjamini–Hochberg method was used for multiple‐comparison correction in both paired comparisons and post‐matching multivariable regression analyses [34], and adjusted *q* values were reported. Multivariable regression models were further constructed in the matched samples. SHG, AF, SAAID, and ELCOR were used as dependent variables, and exercise group was used as the independent variable. Age, BMI, smoking, alcohol use, and average daily sleep duration were adjusted to reduce potential residual confounding. Smoking and alcohol use were included as binary categorical variables, while age, BMI, and average daily sleep duration were included as continuous variables. Standard errors were clustered at the matched‐pair level. Human statistical analyses were performed using Python 3.14.2 (Python Software Foundation).

## 3 Results

### 3.1 Exercise Shifted Two‐Photon Features of Middle‐Aged Mouse Skin Toward a Younger State

During the 4 weeks intervention, the average daily running distance of the ME mice was 8.37 ± 2.78 km/day, corresponding to a cumulative running distance of 234.41 ± 77.82 km per mouse over the 4 weeks intervention period. Two‐photon imaging of mouse dorsal skin showed that, after exercise intervention, the ME group had stronger AF and SHG signals than the MS group. The fibers appeared clearer and more continuous, with fewer local areas of shrinkage, shortening, or discontinuity. Overall morphology in the ME group was closer to that in the YS group than to that in the MS group (Figure 1A).

**FIGURE 1:**
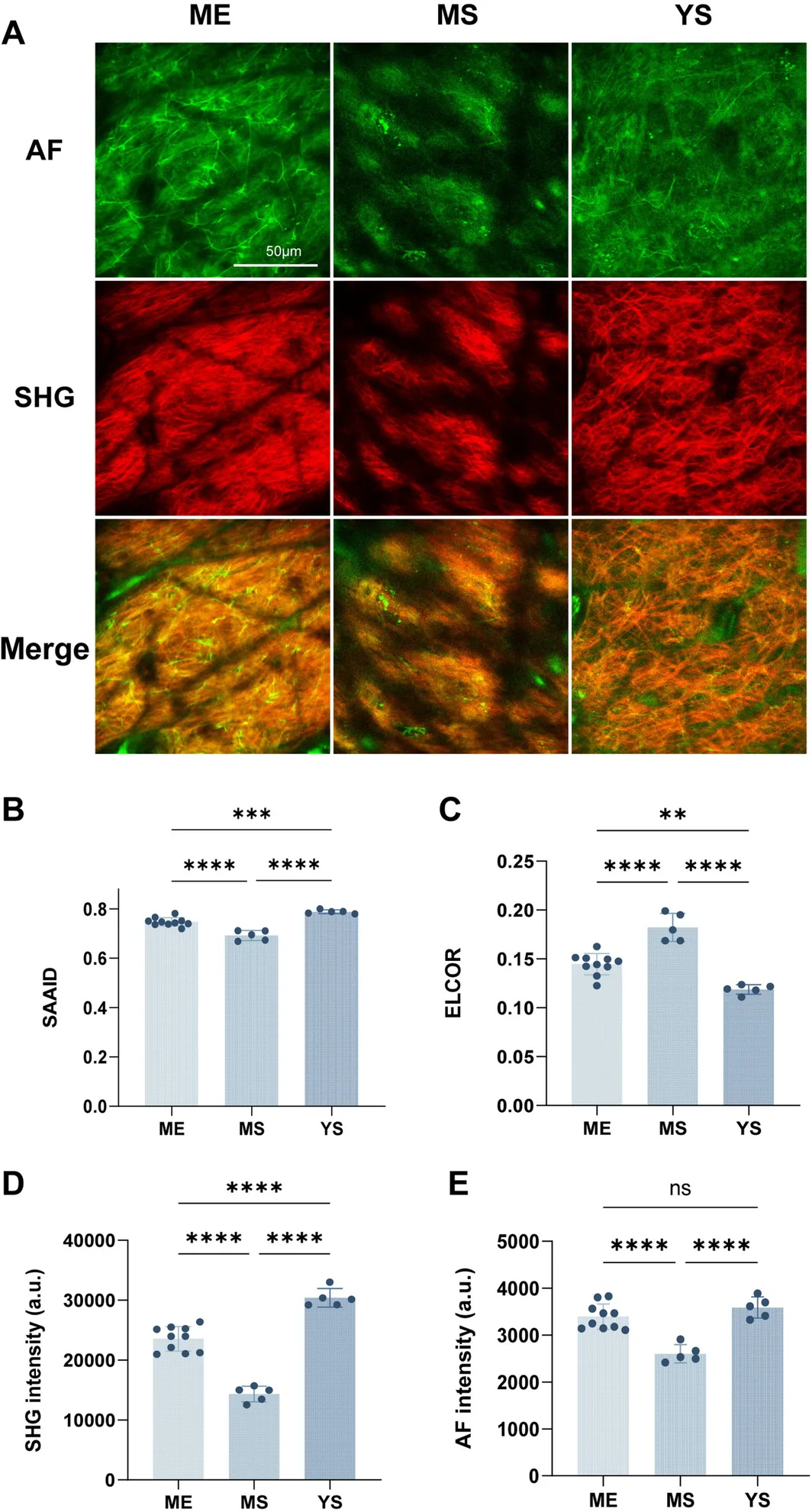
Two‐photon imaging of mouse dorsal skin. (A) Representative two‐photon images of dorsal skin from ME, MS, and YS mice. The AF channel is shown in green and represents elastic fiber‐related autofluorescence signals. The SHG channel is shown in red and represents collagen‐related second harmonic generation signals. The merged image combines AF and SHG signals and shows their spatial distribution. Scale bar: 50 μm. (B–E) Quantitative analysis of SAAID, ELCOR, SHG, and AF. Quantitative results are shown as mean ± standard deviation, *n* = 10 (ME), *n* = 5 (MS, YS). Between‐group comparisons were performed using one‐way ANOVA followed by Tukey's multiple‐comparison test. *p < 0.05, **p < 0.01, ***p < 0.001, ****p < 0.0001; ns indicates no statistically significant difference.

SAAID values in the ME, MS, and YS groups were 0.748 ± 0.017, 0.692 ± 0.020, and 0.788 ± 0.008, respectively. The ME group showed higher SAAID than the MS group but slightly lower SAAID than the YS group (both *p* < 0.001; Figure 1B). ELCOR values in the three groups were 0.145 ± 0.011, 0.182 ± 0.014, and 0.119 ± 0.005, respectively. The ME group had lower ELCOR than the MS group but still higher ELCOR than the YS group (both *p* < 0.01; Figure 1C). SHG values in the ME, MS, and YS groups were 23 568 ± 2044, 14 345 ± 1294, and 30 404 ± 1556, respectively. Between‐group differences for SHG followed the same pattern as for SAAID, with higher SHG in the ME group than in the MS group but lower SHG than in the YS group (both *p* < 0.0001; Figure 1D). AF values in the three groups were 3395 ± 272.5, 2603 ± 194.0, and 3587 ± 225.2, respectively. AF was higher in the ME group than in the MS group (*p* < 0.0001), with no significant difference between the ME and YS groups (*p* = 0.3462; Figure 1E).

These results indicate that exercise intervention can partly improve age‐related abnormalities in two‐photon indicators related to dermal collagen and elastic fibers in middle‐aged mice, with multiple indicators in the ME group shifting toward those of the young sedentary group.

### 3.2 Immunofluorescence Confirmed That Exercise Improved Collagen and Elastic Fiber Structure in Middle‐Aged Mouse Skin

Immunofluorescence staining for Collagen I and Elastin was performed on mouse dorsal skin to validate the two‐photon results (Figure 2A). Compared with the MS group, the ME group showed stronger Elastin and Collagen I signals, a larger positive signal coverage area, and more continuous fiber structures. Local breakage and sparse changes were reduced, and the overall pattern approached that observed in the YS group (Figure 2A).

**FIGURE 2:**
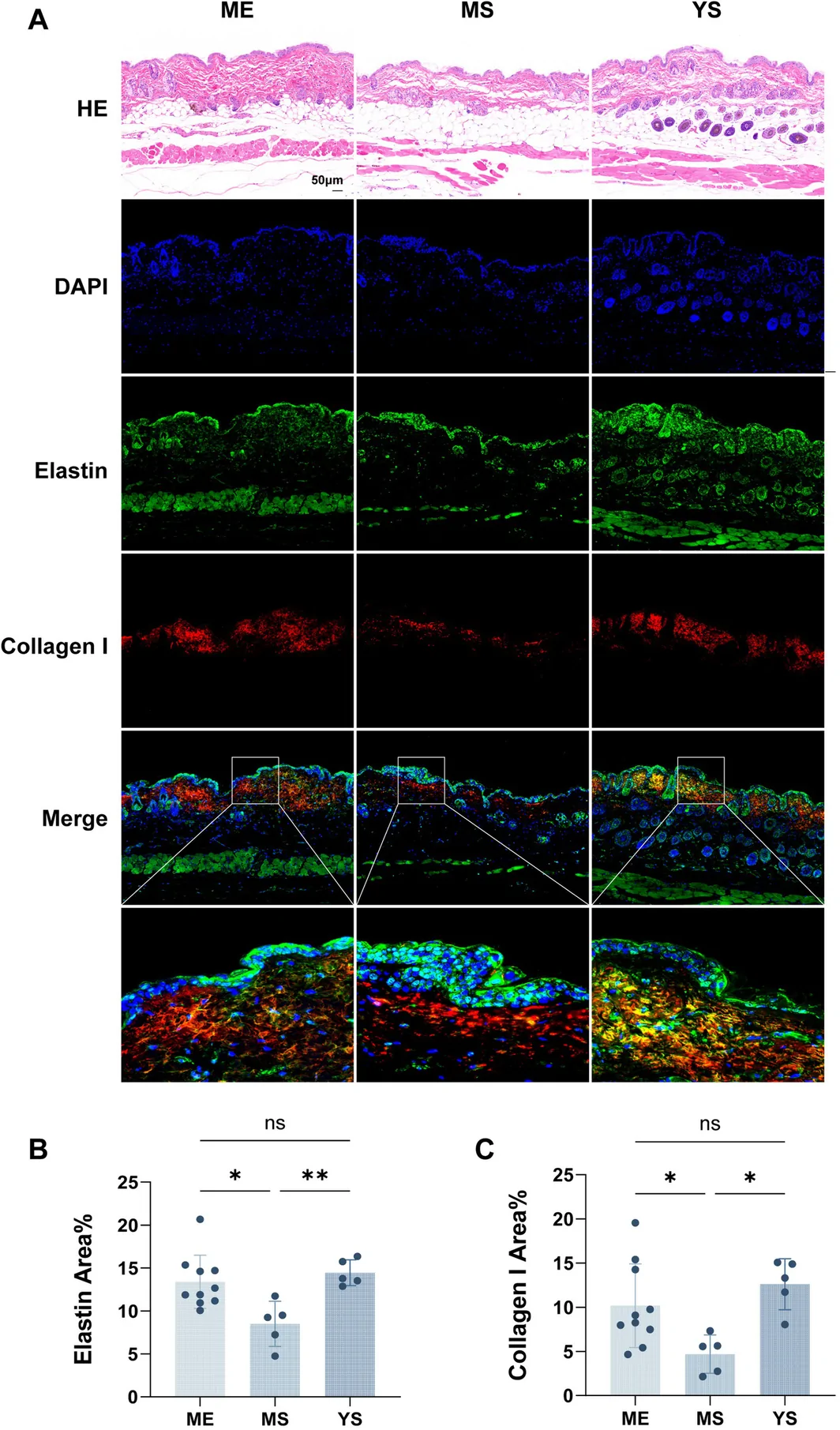
HE staining and immunofluorescence staining of mouse dorsal skin. (A) Representative HE staining and immunofluorescence images of dorsal skin from ME, MS, and YS mice. DAPI is shown in blue for nuclei, Elastin is shown in green in the FITC channel, and Collagen I is shown in red in the Cy3 channel. The merged image combines DAPI, Elastin, and Collagen I signals, and the enlarged images below show local dermal fiber regions. Scale bar: 50 μm (applies to all images except the enlarged panels). (B–C) Quantitative analysis of Elastin Area% and Collagen I Area%, reflecting the positive signal area proportions of Elastin and Collagen I, respectively. Data are shown as mean ± standard deviation, *n* = 5 per group. Between‐group comparisons were performed using one‐way ANOVA followed by Tukey's multiple‐comparison test. *p < 0.05, **p < 0.01; ns indicates no statistically significant difference.

Elastin Area% values in the ME, MS, and YS groups were 13.39 ± 3.11, 8.49 ± 2.63, and 14.45 ± 1.51, respectively. The ME group showed higher Elastin Area% than the MS group (*p* = 0.0108) and was close to the YS group, with no significant difference between the ME and YS groups (*p* = 0.7583; Figure 2B). Collagen I Area% values in the ME, MS, and YS groups were 10.18 ± 4.74, 4.69 ± 2.17, and 12.61 ± 2.90, respectively. The ME group showed higher Collagen I Area% than the MS group (*p* = 0.0473) and was close to the YS group, with no significant difference between the ME and YS groups (*p* = 0.5013; Figure 2C).

The immunofluorescence results were generally consistent with the two‐photon results. Quantitative analysis showed that signals related to Collagen I and Elastin increased in the ME group compared with the MS group, and no statistically significant differences were detected between the ME and YS groups. In the representative merged images, the ME group showed improved fiber‐related staining compared with the MS group, although some differences in fiber distribution and tissue organization remained visible compared with the YS group.

### 3.3 Characteristics of Human Participants and Propensity Score Matching Results

A total of 156 female volunteers were initially enrolled in the human study. The sample size was determined with reference to relevant published clinical observational studies and was considered sufficient for the planned propensity score matching and statistical analyses. After screening according to inclusion and exclusion criteria and image quality control, no participants were excluded, and all 156 participants were included in subsequent statistical analyses. All participants completed facial two‐photon detection and the exercise questionnaire. According to the prespecified analysis plan, human data were processed using both the 150 min/week and median‐based grouping strategies. The distribution of propensity scores and sample size changes is shown in Figure S1.

In the 150 min/week grouping strategy, the high‐ and low‐exercise groups included 48 and 108 participants before matching, respectively. After propensity score matching using age as the covariate, 48 matched pairs were obtained for subsequent comparisons. After matching, the age difference between groups was markedly reduced, with the most obvious improvement in balance (Table 1). BMI, smoking, alcohol use, and average daily sleep duration still showed some residual differences.

**TABLE 1:** Baseline characteristics before and after age‐based propensity score matching under the two exercise grouping strategies.

Variable	Pre‐match high	Pre‐match low	SMD	Post‐match high	Post‐match low	SMD
150 min/week grouping (pre‐match: *n* = 48 vs. *n* = 108; post‐match: *n* = 48 vs. *n* = 48)						
Age, years	30.67 ± 9.62	33.01 ± 9.44	0.246	30.67 ± 9.62	30.75 ± 9.59	0.009
BMI, kg/m^2^	20.83 ± 2.09	20.88 ± 2.13	0.025	20.83 ± 2.09	21.11 ± 2.21	0.133
Average daily sleep duration, h	7.65 ± 0.87	7.09 ± 0.93	0.620	7.65 ± 0.87	7.09 ± 1.08	0.565
Smoking, *n* (%)						
No	48 (100.0)	107 (99.1)		48 (100.0)	48 (100.0)	
Yes	0 (0.0)	1 (0.9)	0.137	0 (0.0)	0 (0.0)	N/A
Alcohol use, *n* (%)						
No	41 (85.4)	83 (76.9)		41 (85.4)	35 (72.9)	
Yes	7 (14.6)	25 (23.1)	0.220	7 (14.6)	13 (27.1)	0.312
Median‐based grouping (pre‐match: *n* = 73 vs. *n* = 83; post‐match: *n* = 69 vs. *n* = 69)						
Age, years	30.81 ± 9.52	33.59 ± 9.40	0.294	31.28 ± 9.55	31.54 ± 8.94	0.028
BMI, kg/m^2^	20.88 ± 2.20	20.85 ± 2.05	0.017	20.96 ± 2.20	20.81 ± 2.06	0.070
Average daily sleep duration, h	7.49 ± 0.98	7.06 ± 0.87	0.460	7.49 ± 1.00	7.09 ± 0.87	0.433
Smoking, *n* (%)						
No	73 (100.0)	82 (98.8)		69 (100.0)	68 (98.6)	
Yes	0 (0.0)	1 (1.2)	0.156	0 (0.0)	1 (1.4)	0.171
Alcohol use, *n* (%)						
No	63 (86.3)	61 (73.5)		59 (85.5)	50 (72.5)	
Yes	10 (13.7)	22 (26.5)	0.324	10 (14.5)	19 (27.5)	0.324

*Note:* Continuous variables are presented as mean ± standard deviation, and categorical variables as *n* (%). Propensity score matching used age as the matching covariate and was performed using 1:1 nearest‐neighbor matching without replacement. SMD was used to assess covariate balance between groups; |SMD| < 0.1 is generally considered to indicate good balance. For categorical variables, SMD is displayed in the “Yes” category row. If no events occurred in either group after matching, SMD could not be calculated and was recorded as N/A.

Abbreviations: AF, autofluorescence; BMI, body mass index; Cy3, cyanine 3; DAPI, 4′,6‐diamidino‐2‐phenylindole; ELCOR, elastin‐to‐collagen ratio; FITC, fluorescein isothiocyanate; ME, middle‐aged exercise group; MS, middle‐aged sedentary group; SAAID, SHG‐to‐AF aging index of dermis; SHG, second harmonic generation; SMD, standardized mean difference; YS, young sedentary group.

In the median‐based grouping strategy, the cohort median WeeklyMinutes value of 30 min/week was used as the cutoff, and participants were divided into the above‐median group and the at‐or‐below‐median group. Before matching, the two groups included 73 and 83 participants, respectively. After the same age‐based propensity score matching method, 69 matched pairs were obtained. Results under this grouping strategy were similar to those under the 150 min/week grouping strategy. The age difference was also markedly reduced, while some other covariates remained incompletely balanced.

Under both grouping strategies, between‐group comparability improved after age matching. Considering that some lifestyle variables still showed residual differences, BMI, smoking, alcohol use, and average daily sleep duration were further included in subsequent regression adjustment.

### 3.4 Matched Two‐Photon Results in the Primary 150 Min/Week Analysis

In the primary 150 min/week analysis, comparisons were performed using 48 matched pairs after propensity score matching. Representative images showed that the high‐exercise group had generally stronger AF and SHG signals, more orderly fiber arrangement, more continuous distribution, and greater fiber abundance. In contrast, the low‐exercise group showed relatively weaker signals, looser fiber arrangement, and poorer local structural integrity (Figure 3A).

**FIGURE 3:**
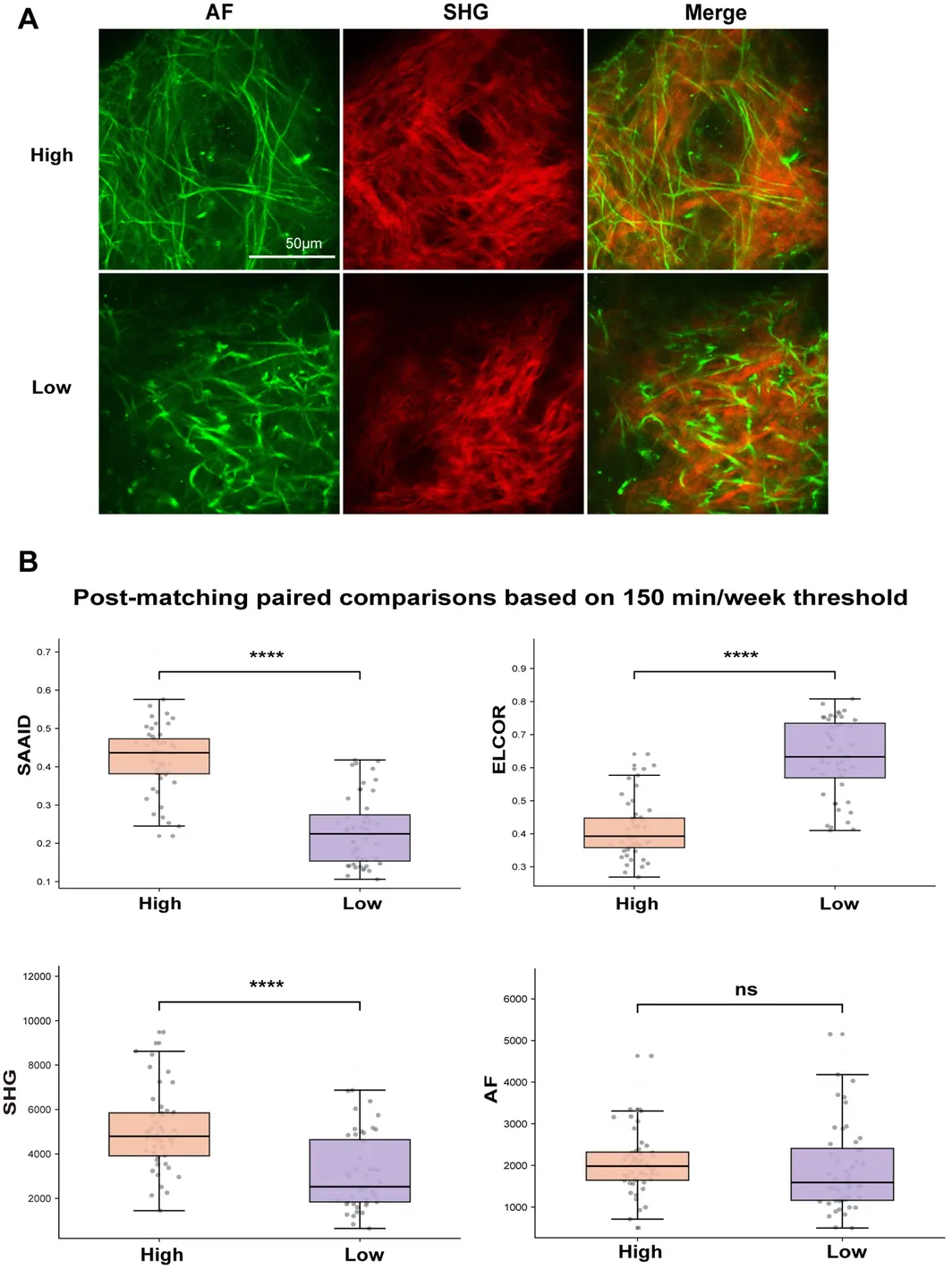
Facial skin two‐photon imaging and quantitative analysis under the 150 min/week grouping strategy. (A) Representative two‐photon images of right zygomatic facial skin from the high‐ and low‐exercise groups. The AF channel is shown in green and represents elastic fiber‐related autofluorescence signals. The SHG channel is shown in red and represents collagen‐related second harmonic generation signals. The merged image combines AF and SHG signals and shows their spatial distribution. Scale bar: 50 μm. (B) Paired comparison results of SAAID, ELCOR, SHG, and AF after age‐based propensity score matching, with 48 matched pairs. Paired *t*‐tests or Wilcoxon signed‐rank tests were used according to the distribution of paired differences. Multiple comparisons were corrected using the Benjamini–Hochberg method. *q < 0.05, **q < 0.01, ***q < 0.001, ****q < 0.0001; ns indicates no statistically significant difference.

After matching (Figure 3B), SAAID was 0.420 ± 0.085 in the high‐exercise group and 0.233 ± 0.091 in the low‐exercise group. SHG values were 5060.655 ± 1814.730 and 3115.486 ± 1654.403, respectively, both higher in the high‐exercise group (both *q* < 0.001). ELCOR was 0.414 ± 0.088 in the high‐exercise group and 0.630 ± 0.116 in the low‐exercise group, with a lower value in the high‐exercise group (*q* < 0.001). AF values were 2059.365 ± 761.685 and 1915.476 ± 1034.732 in the two groups, respectively, with no statistically significant difference (*q* = 0.385).

Under the 150 min/week grouping strategy, a higher exercise level was associated with higher SAAID and SHG, lower ELCOR, and no obvious change in AF. Taken together, these indicator changes suggest that higher exercise levels are associated with better dermal fiber status in facial skin.

### 3.5 Supplementary Analysis Based on the Median Grouping Strategy

In the supplementary median‐based analysis, comparisons were performed using 69 matched pairs after propensity score matching. The changes in facial two‐photon indicators between the high‐ and low‐exercise groups were generally consistent with those in the primary 150 min/week analysis. Representative images showed a more consistent fiber orientation, more continuous strand‐like connections, and a more coherent overall texture in the high‐exercise group. In the low‐exercise group, fibers were more disorganized, with visible local interruptions and fragmentation, poorer continuity, and generally weaker signals in the merged images (Figure 4A).

**FIGURE 4:**
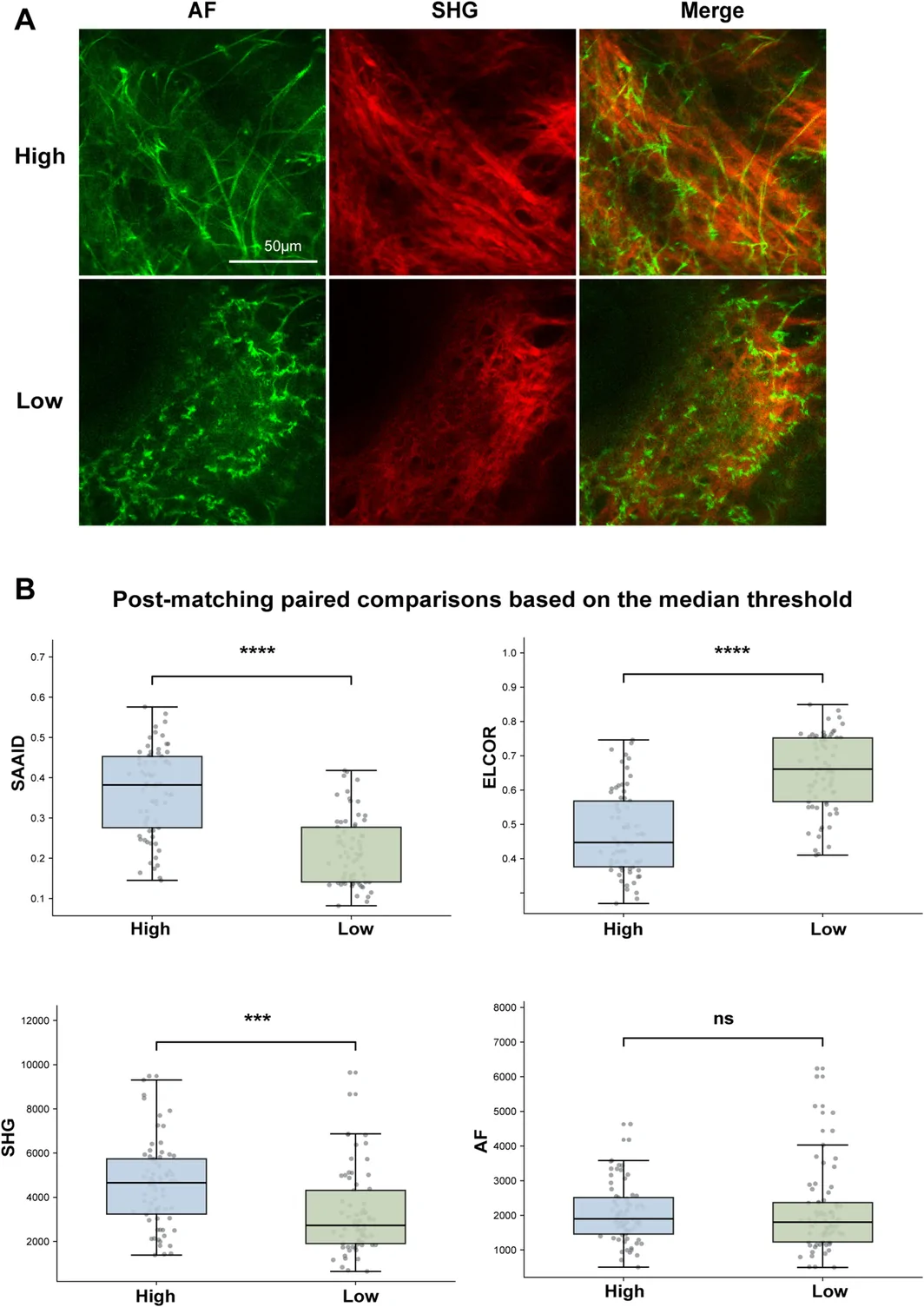
Facial skin two‐photon imaging and quantitative analysis under the median‐based grouping strategy. (A) Representative two‐photon images of right zygomatic facial skin from the high‐ and low‐exercise groups. The AF channel is shown in green and represents elastic fiber‐related autofluorescence signals. The SHG channel is shown in red and represents collagen‐related second harmonic generation signals. The merged image combines AF and SHG signals and shows their spatial distribution. Scale bar: 50 μm. (B) Paired comparison results of SAAID, ELCOR, SHG, and AF after age‐based propensity score matching, with 69 matched pairs. Paired *t*‐tests or Wilcoxon signed‐rank tests were used according to the distribution of paired differences. Multiple comparisons were corrected using the Benjamini–Hochberg method. *q < 0.05, **q < 0.01, ***q < 0.001, ****q < 0.0001; ns indicates no statistically significant difference.

Quantitative analysis showed that SAAID and SHG were higher in the high‐exercise group than in the low‐exercise group, with values of 0.365 ± 0.109 versus 0.216 ± 0.086 and 4547.563 ± 1927.049 versus 3262.500 ± 1875.309, respectively (*q* < 0.001 and *q* < 0.001; Figure 4B). ELCOR was lower in the high‐exercise group than in the low‐exercise group, with values of 0.475 ± 0.121 and 0.653 ± 0.113, respectively (*q* < 0.001). AF values were similar between the two groups, 2070.991 ± 855.999 and 2077.018 ± 1212.291, with no statistically significant difference (*q* = 0.971).

### 3.6 Post‐Matching Multivariable Regression Adjustment

In the age‐matched samples, multivariable regression analyses were further performed for SAAID, ELCOR, SHG, and AF, adjusting for age, BMI, smoking, alcohol use, and average daily sleep duration, with cluster‐robust standard errors estimated at the matched‐pair level (Figure 5).

**FIGURE 5:**
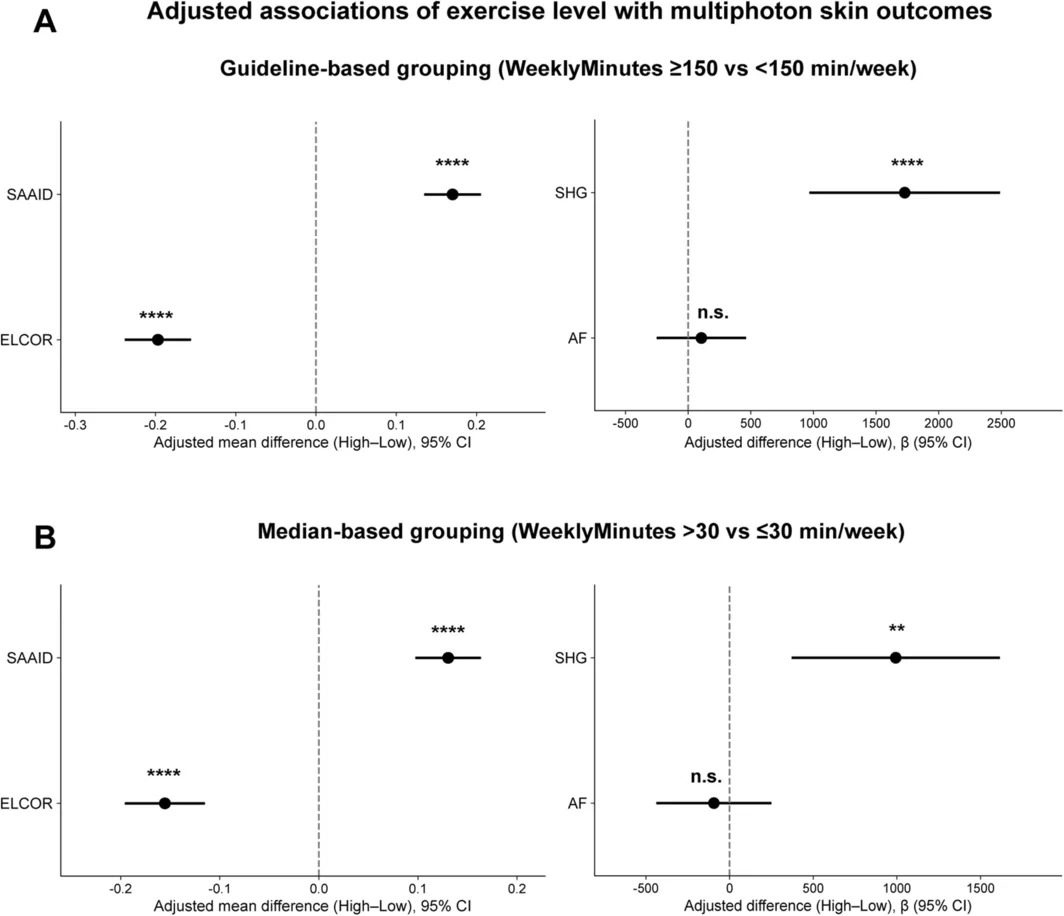
Post‐matching multivariable regression analysis. (A) Adjusted associations between higher exercise level and SAAID, ELCOR, SHG, and AF under the 150 min/week grouping strategy. (B) Adjusted associations between higher exercise level and SAAID, ELCOR, SHG, and AF under the median‐based grouping strategy. Points indicate adjusted mean differences in the high‐exercise group relative to the low‐exercise group; horizontal lines indicate 95% confidence intervals, and the dashed vertical line indicates an effect value of 0. Models were adjusted for age, BMI, smoking, alcohol use, and average daily sleep duration, with standard errors clustered at the matched‐pair level. The *p*‐values were corrected using the Benjamini–Hochberg method and are shown as *q* values. *q < 0.05, **q < 0.01, ***q < 0.001, ****q < 0.0001; ns indicates no statistically significant difference.

In the 150 min/week grouping strategy, higher exercise level was associated with increased SAAID (β = 0.170, 95% CI, 0.135–0.206; *q* < 0.001), decreased ELCOR (*β* = −0.197, 95% CI, −0.238 to −0.156; *q* < 0.001), and increased SHG (*β* = 1729.369, 95% CI, 968.216–2490.522; *q* < 0.001). AF showed no statistically significant difference (*β* = 105.396, 95% CI, −251.338–462.131; *q* = 0.563; Figure 5A).

The median‐based grouping results were similar. Higher exercise level remained associated with increased SAAID (*β* = 0.131, 95% CI, 0.097–0.164; *q* < 0.001), decreased ELCOR (*β* = −0.155, 95% CI, −0.196 to −0.115; *q* < 0.001), and increased SHG (*β* = 993.275, 95% CI, 370.874–1615.676; *q* = 0.002). AF still showed no statistically significant difference (*β* = −93.810, 95% CI, −437.342–249.722; *q* = 0.592; Figure 5B).

After adjustment, the association directions for SAAID, SHG, and ELCOR remained unchanged under both grouping strategies, while AF showed no statistically significant difference in either analysis.

## 4 Discussion

### 4.1 Main Findings

This study obtained consistent results from animal experiments, histological validation, and noninvasive facial detection in humans. In mice, exercise‐related changes were reflected by both two‐photon imaging and histological staining. In humans, the results were consistent across the two grouping strategies. Differences in exercise level were reflected in two‐photon indicators related to skin collagen and elastic fibers, providing both experimental and population‐based support for the use of two‐photon microscopy in noninvasive evaluation of human facial skin.

### 4.2 Interpretation of Two‐Photon Indicator Changes and Histological Support

In this study, exercise‐related differences were mainly observed in SHG, SAAID, and ELCOR, whereas AF showed less consistent changes in the human component. This suggests that the effect of exercise on skin fiber status may be more evident in collagen‐related structures and in the overall balance between collagen and elastic fibers, rather than as an isolated change in a single elastic fiber‐related fluorescence signal.

SHG mainly reflects collagen fiber‐related signals. In both mice and humans, higher SHG indicates better preservation of dermal collagen‐related signals. SAAID is a composite indicator reflecting the relative relationship between collagen‐ and elastic fiber‐related signals and is more sensitive to overall changes in fiber status. AF in the dermis mainly reflects elastic fiber‐related autofluorescence signals. A decrease in AF should be interpreted cautiously, as it may indicate weakened elastic fiber‐related autofluorescence, suggesting possible reduction or degeneration of elastic fibers with aging [35]. However, AF signals in human facial skin may be affected by baseline skin status, local photoaging, dermal depth, and differences in endogenous fluorescence sources, so AF alone should be interpreted with caution [36, 37]. The difference in the clarity of fiber‐like structures in the AF channel between mouse dorsal skin images and human facial skin images should also be understood in relation to sample source and imaging conditions. The two types of samples differ in species, anatomical site, skin thickness, distribution of hair follicles and skin appendages, endogenous fluorescent components, and stable imaging depth, all of which may affect the visual appearance of elastic‐fiber‐related signals in the AF channel. Compared with mouse dorsal skin, human facial skin is thicker and provides a relatively broader dermal imaging range, making clearer fiber‐like structures in the AF channel more readily observable within the selected analysis window. During the analysis, the mouse and human datasets were assigned analysis windows separately according to their respective skin structural characteristics and imaging signal stability, and quantitative analysis was performed under consistent image quality criteria and analysis workflows within each dataset. Raw AF signal intensities were not directly compared between the two types of samples. ELCOR reflects the relative ratio of elastic fiber‐related signals to collagen‐related signals. Changes in this indicator do not simply indicate an increase or decrease in one fiber type; rather, they reflect whether the dermal fiber composition is balanced. The decrease in ELCOR after exercise or in participants with higher exercise levels suggests that this balance may be partly improved.

The mouse histological results provided important support for the above interpretation. Immunofluorescence staining for Collagen I and Elastin showed that, compared with the MS group, collagen‐ and elastin‐related signals were increased in the ME group and quantitatively approached those in the YS group. This was consistent with the direction of the two‐photon results, suggesting that the changes in imaging signals were accompanied by corresponding alterations in dermal fiber structure. It should be noted that the absence of statistically significant differences between the ME and YS groups in the quantitative immunofluorescence analysis does not mean that the skin fiber structure of all mice in the ME group had been completely restored to a young level. In the representative merged images, some differences in fiber distribution and tissue organization could still be observed between the ME and YS groups, which may be related to inter‐individual differences in the degree of improvement after exercise intervention.

Regarding the two immunofluorescence markers, both Elastin and Collagen I showed exercise‐related improvement trends, but their staining patterns and spatial distributions were not completely identical. Elastin signals showed a relatively broad and continuous fiber‐like or network‐like distribution, whereas Collagen I signals were more concentrated in dermal collagen‐rich regions and appeared as relatively localized band‐like or patchy staining. In the MS group, both signals were reduced, with the decrease in Collagen I signals appearing more prominent in the representative images. In the ME group, both signals were increased compared with the MS group and quantitatively approached those in the YS group. The merged images showed that Elastin and Collagen I signals were spatially close in some areas but did not completely overlap, indicating that these two fiber systems differ in their spatial distribution and organization within the dermis. Overall, Collagen I and Elastin changed in the same direction after exercise intervention, but the two markers still differed in staining pattern, spatial distribution, and relative degree of change.

### 4.3 Potential Significance of Exercise in Skin Rejuvenation

Compared with skin rejuvenation strategies that mainly rely on local treatment, exercise is low‐cost, sustainable, and provides systemic health benefits. It is more compatible with daily life and easier to maintain over the long term. Whether exercise can affect deep skin fiber status has long lacked objective and direct evidence [38]. The present results suggest that the effects of exercise are not limited to improvements in metabolism and physical fitness, but may also be related to maintenance of the dermal microenvironment. The two‐photon results provide more direct imaging evidence for the relationship between exercise and skin structure and appearance. Although exercise cannot replace existing aesthetic therapies, its potential to counteract age‐related collagen and elastin degradation may support its use as an adjunctive lifestyle strategy in skin rejuvenation management. The current human evidence is mainly derived from observational analyses and cannot directly establish causality or define the optimal exercise type and dose. The effects of different exercise types, intensities, and durations on dermal collagen and elastic fiber status require further study.

### 4.4 Strengths and Limitations

A major strength of this study is the use of a relatively complete research pathway to evaluate exercise‐related changes in dermal fiber status. The study first used an animal exercise model to observe changes in two‐photon indicators and then validated the imaging findings with Collagen I and Elastin immunofluorescence staining. On this basis, the same in vivo two‐photon imaging approach was further applied to human facial skin. Therefore, the human findings were supported by animal‐level histological evidence rather than by imaging observations alone.

The imaging method used in this study also has methodological advantages. Several noninvasive imaging techniques have been used for skin assessment, including reflectance confocal microscopy, optical coherence tomography, and high‐frequency skin ultrasound [39, 40]. These methods provide useful information on skin morphology, epidermal and superficial dermal structures, or dermal thickness and echogenicity. In comparison, two‐photon microscopy combines two‐photon excited fluorescence and second harmonic generation, allowing label‐free visualization of endogenous fluorescence signals and collagen‐related SHG signals in vivo [19, 41, 42]. Therefore, it can provide more direct information on dermal fiber‐related structures. The innovation of the present study lies in combining animal intervention, histological validation, and human facial two‐photon imaging to evaluate exercise‐related changes in collagen‐ and elastic fiber‐related indicators.

Another strength of this study is that the human analysis used both 150 min/week and median‐based grouping strategies, and the conclusions were consistent between the two approaches. Imaging site, image screening, ROI selection, and subsequent analysis were all performed according to unified rules to reduce subjective bias.

This study also has limitations. The animal experiment used an endpoint between‐group comparison design, and longitudinal two‐photon imaging before and after exercise intervention was not performed in the same mice. Thus, dynamic within‐animal changes in skin structure during the exercise intervention could not be directly evaluated. Future studies could take advantage of the noninvasive nature of two‐photon imaging to perform repeated measurements in the same animals before and after intervention, further validating the longitudinal effects of exercise on dermal collagen and elastic fiber structures.

The animal component used a voluntary wheel running model, which can better mimic spontaneous physical activity. However, the effects of exercise intervention are not limited to exercise volume itself and may also be accompanied by systemic changes in body weight, food intake, metabolic status, inflammatory status, hormonal state, and stress response. Running distance was recorded in the exercise group, but these related parameters were not further measured. The observed two‐photon imaging parameters and histological changes are more appropriately interpreted as being associated with voluntary wheel running, while indirect effects of exercise‐related systemic physiological changes cannot be completely excluded. Future studies incorporating systemic physiological measurements during exercise intervention may help further clarify the independent role of exercise in dermal fiber remodeling.

The human component was cross‐sectional and observational, so the current results are more appropriately interpreted as associations and cannot directly infer causality. To reduce the influence of confounding factors, BMI, smoking, alcohol use, and average daily sleep duration were further adjusted for in the age‐matched samples. These covariates were all derived from questionnaire data, and residual bias from unmeasured confounders and measurement error may exist. Because the sample size was limited and some covariate information was incomplete, multiple combined matching strategies were not performed. Instead, multivariable regression adjustment was conducted after age matching. The number of covariates included in the adjustment was limited. Other factors that may affect the results, including sun exposure, skincare habits, dietary structure, and occupational exposure, were not investigated, mainly because these factors currently lack universally recognized quantitative evaluation criteria. The human sample consisted only of female volunteers, so the results apply mainly to this specific population. Whether the same pattern exists in different sexes and in populations with different baseline skin conditions requires further study.

Human exercise exposure was calculated from self‐reported questionnaires. Although grouping was based on guideline‐recommended amounts, classification of exercise intensity remained relatively crude. The duration of regular exercise was not further collected, and the time interval between the most recent exercise session and two‐photon examination was not recorded. The differences in two‐photon imaging parameters observed in the human component therefore cannot clearly distinguish chronic adaptations to long‐term regular exercise from recent exercise effects, or the combined influence of both. Because the human grouping was based on daily exercise habits and total weekly exercise duration rather than immediate assessment after a single exercise session, the relevant findings are more appropriately interpreted as skin status differences associated with long‐term or relatively stable exercise habits. Future studies could incorporate information on exercise duration, exercise type, exercise intensity, and the timing of the most recent exercise session before examination, and could adopt a longitudinal follow‐up design to better distinguish long‐term chronic adaptations from short‐term exercise effects.

## 5 Conclusion

In conclusion, this study obtained consistent findings from mouse experiments, histological validation, and noninvasive human facial detection, suggesting that exercise may favorably affect dermal collagen and elastic fibers. Supported by animal‐level histological evidence, two‐photon microscopy can directly and objectively reflect exercise‐related changes in skin fibers and may have potential value for noninvasive assessment of human facial skin. At this stage, the evidence supports an associative rather than causal interpretation and also indicates the translational potential of this technique. Future studies with larger samples, longitudinal follow‐up, and intervention designs are needed to further clarify the effects of different exercise modes and doses on skin fiber changes. If these findings are further validated, exercise may become an effective adjunctive strategy for skin rejuvenation management.

## Author Contributions

Qi Luo contributed to investigation, data collection, data curation, formal analysis, visualization, and drafting of the manuscript. Minghao Wang contributed to data collection, data organization, and manuscript revision. Zhu Shen contributed to study conception and design, methodology, supervision, project administration, and critical revision of the manuscript. All authors read and approved the final manuscript.

## Funding

This work was supported by the Scientific Research Matching Fund of Guangdong Provincial People's Hospital (No. KY0120220452).

## Ethics Statement

This study was approved by the Ethics Committee of Guangdong Provincial People's Hospital on February 13, 2026 (approval No. KY2026‐119‐01). All human participants provided written informed consent before enrollment. All animal procedures were conducted in accordance with institutional guidelines for the care and use of laboratory animals.

## Consent

The authors have nothing to report.

## Conflicts of Interest

The authors declare no conflicts of interest.

## Supporting information


**Figure S1:** Propensity score distribution and sample size changes before and after matching under the two exercise grouping strategies. (A) Propensity score distribution before and after matching under the 150 min/week grouping strategy. (B) Propensity score distribution before and after matching under the median‐based grouping strategy. (C) Sample size changes before and after matching under the 150 min/week grouping strategy. (D) Sample size changes before and after matching under the median‐based grouping strategy.

**Video S1:** Representative mouse exercise activity during the 4 weeks voluntary wheel running intervention.

## Data Availability

The data that support the findings of this study are available on request from the corresponding author. The data are not publicly available due to privacy or ethical restrictions.
